# Khat consumption and undernutrition among adult population in Ethiopia: A systematic review and meta-analysis

**DOI:** 10.1371/journal.pone.0299538

**Published:** 2024-04-23

**Authors:** Abdu Oumer

**Affiliations:** School of Public Health, College of Medicine and Health Sciences, Haramaya University, Harar, Ethiopia; Wachemo University, INDIA

## Abstract

**Background:**

In Ethiopia, malnutrition is a public health threat causing a significant burden of morbidity, mortality, and economic crisis. Simultaneously, khat consumption is alarmingly increasing among adults, yet it might contribute to the existing burden of malnutrition, where the current evidence is inconclusive. Hence, this review was to estimate the association between khat consumption and undernutrition among adults in Ethiopia.

**Methods:**

A comprehensive search for Google, Google Scholar, and PubMed, coupled with a thorough manual search of the literature, was done up to date, October 18, 2023, using relevant search terms: “impact," "effects," “khat chewing," “khat consumption," "Ethiopia," “nutritional status," and "undernutrition." An updated PRISMA guideline was used to select relevant literature. The extracted data was summarized in narrative summaries, descriptions, and meta-analyses. The risk of bias was assessed. The results are presented in forest plots and funnel plots to assess publication bias. A pooled effect size (odds ratio) with a 95% certainty level was reported.

**Results:**

While a total of 17 articles (n = 45,679) were included in the narrative review, only 15 articles were included in the quantitative meta-analysis. The majority of studies had a low and moderate risk of bias (based on risk of bias assessment tool), mainly due to unclear exposure assessment and high study heterogeneity. A total of 11 studies were cross-sectional studies (71%), three were comparative studies (17.4%), and three were case control studies (17.4%). There is a higher risk of publication bias as evidenced by the funnel plot. Overall, five studies were from the Oromia region, and three studies were conducted at the national level. Overall, chewing had been shown to significantly increase the risk of undernutrition by 53% (pooled OR = 1.53; 95% CI: 1.09–2.16) under a random effect model. Under the fixed effect model, higher weight was given to national-level studies with higher samples, where chewing contributed to a 12% increased risk of undernutrition (AOR = 1.12; 95% CI: 1.01–2.23). Hence, khat chewing could raise the odds of undernutrition by 12–53%.

**Conclusion:**

There is evidence of an association between khat chewing and an increased risk of undernutrition among adults in Ethiopia, which highlights the need for public health interventions to address the potential adverse effects of khat chewing on nutritional status.

## Introduction

Undernutrition remains a significant public health challenge worldwide, particularly in low- and middle-income countries [1,2]. It is a complex issue influenced by various factors, including dietary habits, socio-economic status, cultural practices, and substance use [3,4]. Among the many substances consumed globally, khat (Catha edulis) has gained attention for its potential impact on nutritional status. Khat is a widely chewed psychoactive plant native to East Africa, including Ethiopia, where its consumption is deeply rooted in social and cultural traditions [5–7]. Given the prevalence of khat chewing and the high burden of undernutrition in Ethiopia, it is crucial to examine the potential relationship between khat chewing and the risk of undernutrition among adults, where there is no clear evidence yet. For instance, studies from Ethiopia showed that the prevalence of khat chewing is about 19–27% [8] and 17% among students [9].

Despite the long-standing practice of khat chewing and the concerning levels of undernutrition in Ethiopia, the specific effects of khat consumption on nutritional status have not been extensively investigated. Most existing studies on khat have primarily focused on its psychoactive properties [10–12] and associated health consequences, such as cardiovascular effects, mental health outcomes, and social implications [11,13,14]. While these studies have contributed valuable insights, the impact of khat chewing on nutritional status remains a relatively understudied area.

The limited number of studies examining the link between khat chewing and undernutrition have yielded mixed findings, ranging from suggestions of adverse nutritional effects to reports of no significant association [15–20]. These conflicting results highlight the urgent need for a comprehensive and systematic evaluation of the existing evidence. For instance, some studies have suggested a potential association between khat chewing and adverse nutritional outcomes, including weight loss, decreased appetite, and poor dietary intake [21,22], which could be attributed to the stimulant properties of khat suppressing appetite, altering metabolism, and reducing food intake [14,15,21,23,24]. However, other studies have reported conflicting results, failing to establish a clear link between khat chewing and undernutrition [18,25,26]. These discrepancies may be due to variations in study design, sample characteristics, and the lack of rigorous synthesis of existing evidence.

Moreover, the existing literature on khat chewing and undernutrition in Ethiopia is limited by several factors. First, the available studies are often small-scale and cross-sectional, limiting their ability to establish causal relationships or provide robust evidence. Second, the methodologies used to assess undernutrition outcomes vary, making it challenging to compare and generalize the findings. Third, the majority of studies have focused on specific populations and localities, which may not fully represent the diverse adult population of Ethiopia. Lastly, the absence of a comprehensive synthesis of the existing evidence through a systematic review and meta-analysis hinders the ability to draw conclusive findings and inform evidence-based interventions.

Limited evidence exists on khat chewing’s impact on adult undernutrition in Ethiopia, despite its prevalence and the country’s significant undernutrition burden. This knowledge gap necessitates a comprehensive systematic review and meta-analysis to synthesize existing research. By systematically reviewing and quantitatively analyzing the literature, this study aims to provide a definitive assessment of the association between khat chewing and undernutrition outcomes. This will inform future research and interventions, benefiting policymakers, public health practitioners, and researchers tackling undernutrition and substance use issues in Ethiopia.

## Materials and methods

### Data sources

The data used for this review article were extracted from a secondary review of existing literature on the association between khat chewing and the odds of undernutrition in Ethiopia. Peer-reviewed journal articles and other relevant, unpublished works from academic repositories were used for the review. The selected articles and unpublished manuscripts were consulted for important data on the association between khat chewing and the risk of undernutrition for adults since 2000.

### Search strategies

A thorough and systematic search was employed for relevant literature in databases. We searched for both published and unpublished studies reporting the association between khat chewing and nutritional status among the adult population in Ethiopia. Exhaustive yet systematic searches were conducted in Google Scholar, PubMed, CINAHL, EMBASE, and MEDLINE using keyword combinations and MeSH terms. A variety of combinations of keywords and mesh terms were tried to come up with exhaustive lists. A more advanced search approach was employed by year of publication and area of publication. In addition, a thorough manual search of the literature was done. The search was up to date as of October 18, 2023.

A search was made using relevant keywords and MeSH terms using Boolean operators. Hence, the search was conducted using the following key words: "impact," "effects," “khat chewing," “khat consumption," "Ethiopia," “nutritional status," and "undernutrition." We employed a variety of combinations of these terms to come up with unbiased search results. The reference list of the included articles was screened thoroughly for additional studies. We have limited studies reported in English, but still, we did not find any relevant studies published other than in English for the Ethiopian context. The search is not restricted to a specific time period. Other databases, like conference proceedings, abstracts, preprints, and articles published as supplements to journals, were included. Additionally, other local university libraries and repositories were also searched for relevant articles on the issue.

### Inclusion of primary studies

This review will consider analytical observational studies, including analytical cross-sectional studies, prospective and retrospective cohort studies, and case-control studies. Studies reporting the unconfounded association between khat chewing and nutritional status in the adult population are considered to be eligible for this review. Hence, studies primarily done with khat chewing as exposure or khat chewing captured as potential confounding variables were eligible for the review. However, those studies reported for children and elders aged above 60 years without information on the effect size or the two-by-two table cell values are excluded.

### Study selection

Following the search, all identified citations were exported to and uploaded into Endnote version 20, where duplicates were removed and further screening was conducted for the titles and abstracts of the potential articles. At this stage, further articles not meeting the inclusion criteria were excluded by two independent reviewers against the inclusion criteria and the full text of these articles was retrieved. Any disagreements that arise between the reviewers at each stage of the selection process will be resolved through discussion or with the involvement of a third reviewer. The results of the search and the study inclusion process are reported and presented in a Preferred Reporting Items for Systematic Reviews and Meta-analyses (PRISMA) flow chart [27].

### Exposure and outcome of the review

The primary exposure of this review is khat chewing or khat consumption, defined as any level of khat chewing or consumption as defined by previous studies. The majority of the studies reported or assessed the previous history and current state of khat chewing. These potential variations, in addition to confounding variables such as food consumption and alcohol drinking status, among others, have the potential to introduce heterogeneity in the estimation of effect sizes and the direction of associations. The primary outcome was the nutritional status of adults, defined as a body mass index (BMI) below 18.5 kg/m^2^ based on the WHO classification or MUAC below 23 cm for lactating women [28–30].

### Assessment of methodological quality and risk of bias

Two reviewers assessed eligible studies for their methodological quality using the standardized JBI Critical Appraisal Checklist for analytical cross-sectional studies [31] and case-control studies [32]. The checklist for cross-sectional studies was composed of eight items that assesses the risk of bias and methodological quality. The checklist for case-control studies has 10 items that are used to gauge group comparability; matching cases and controls, measuring exposure, accounting for confounding variables, exposure period, and analysis.

Each response is evaluated as "yes," "no," "unclear,” or “not applicable.” If "yes" is selected, one point is awarded. Studies earning six or more points will be deemed to be of high quality and included in the review considering the relevance of the criteria and using a 60 percentile as rule of thumb. The quality assessment was carried out independently by two reviewers, and disputes were managed accordingly. A narrative report and statistical tables (S1 and S2 Tables) were used to present the findings of the critical appraisal. To ensure the credibility of the review and meta-analysis, the risk of bias for each study was independently assessed by two reviewers using the JBI tool, and the findings were presented accordingly. To ensure consistency and objectivity, any discrepancies between reviewers were resolved through collaborative discussion or consultation with an independent reviewer.

### Data extraction

Two independent reviewers extracted the necessary data from each study using a predefined Excel spreadsheet. The spread sheet was prepared using the standardized JBI data extraction tools, where specific information regarding the participants, study procedures (design), exposure, outcome, cell values, and effect size estimates were captured. Further contacts and requests for details from the authors of the primary studies were made for missing information and additional data for clarification as deemed appropriate. Any differences between the review authors were settled through conversation or consultation with a third independent party.

### Assessing certainty of evidence

The certainty of the evidence for the role khat chewing plays in undernutrition was evaluated using the Grading of Recommendations, Assessment, Development and Evaluation (GRADE) technique, which was used to grade the certainty of the evidence [33]. Any discrepancies among the reviewers were settled via discussion or by consulting a third reviewer. In cases where further information is needed for clarity, the authors of the papers were consulted by email. Absolute risks for the exposed and unexposed, estimates of odds ratio, and an evaluation of the quality of the evidence based on the risk of bias, directness, heterogeneity, precision, and risk of publication bias of the review results will all be provided where applicable.

### Data synthesis and analysis

The data extracted was in MS Excel and exported to STATA version 14 for meta-analysis. The four cells in the two-by-two epidemiologic table values were extracted and used to calculate the odds ratio as an effect size measure along with 95% confidence intervals. Given the anticipated heterogeneity, statistical analyses will be performed using a random-effects model for meta-analysis [34]. The random-effects model was selected for the current study after careful consideration of the observed heterogeneity among the included studies. The presence of diverse methodologies, sample characteristics, and settings within the studies warranted the use of a random-effects model to account for the inherent variability across them. Hence, by employing this model, the study aimed to obtain a more precise and reliable estimate of the overall effect size, taking into account the expected differences among the studies. This approach acknowledges and accommodates the heterogeneity present in the data, allowing for a more robust analysis and interpretation of the findings in the current study than the fixed effect model.

Subgroup analyses were conducted where there was sufficient data to investigate differences by region, population (general and high-risk populations), and study design. Sensitivity analyses were conducted to test decisions made regarding the analysis model and effect size. Funnel plot was generated for publication bias. The pooled estimates are presented graphically in forest plots along with study weights. A funnel plots were used to assess the possibility of publication bias by comparing the sample size against the effect size or standard error of the effect size measure. Statistical tests for funnel plot asymmetry (Egger test, Begg test, and Harbord test) will be performed where appropriate [35]. A narrative synthesis will be conducted for outcomes that were not suitable for pooling into the meta-analysis.

## Results

### Search results

**Fig 1:**
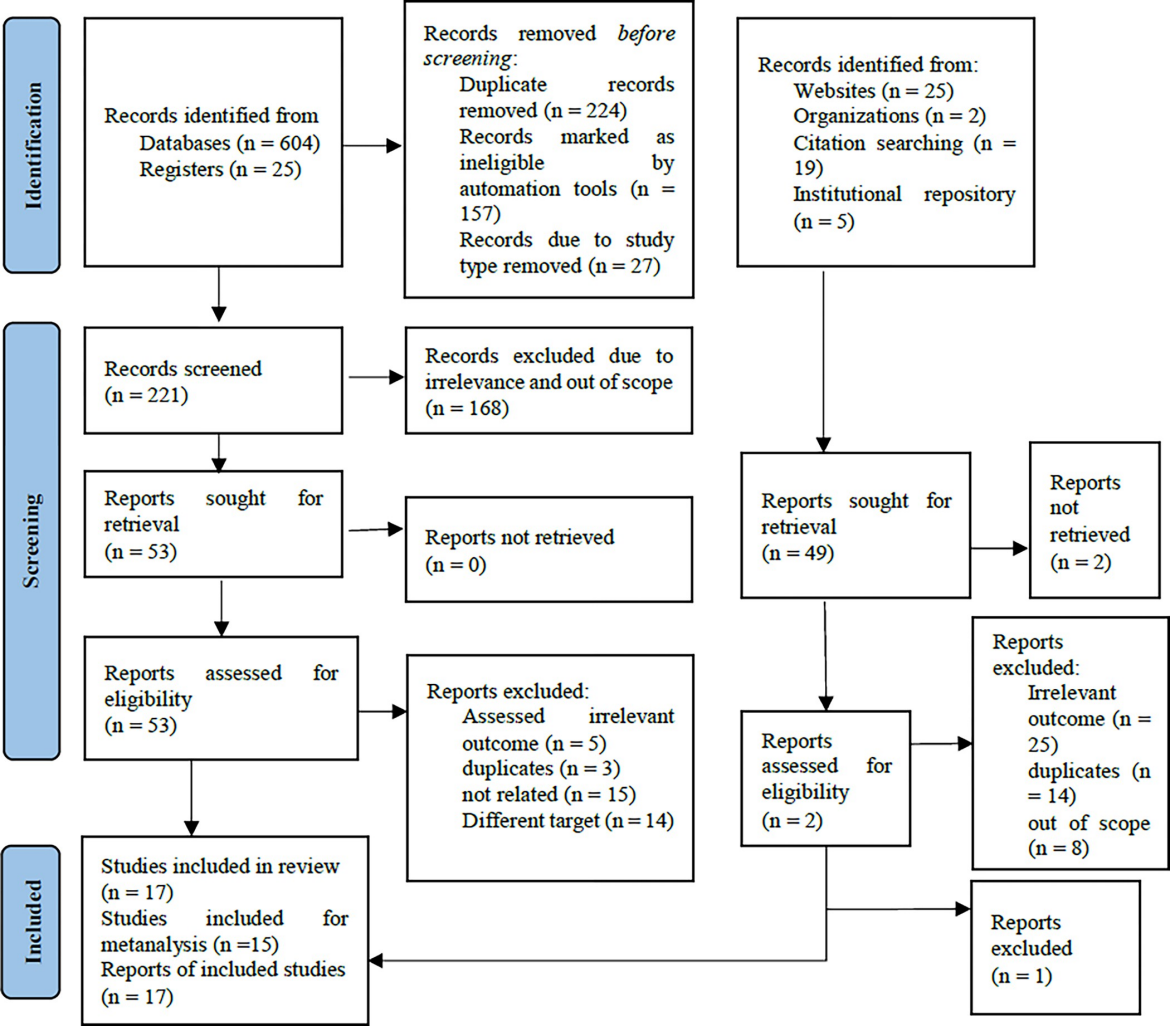
PRISMA flow chart showing the sequential selection of articles for systematic review and meta-analysis.

A total of 604 articles were obtained via database search, and 25 articles from registers were obtained using systematic search. Through our PubMed searches, we found n = 293 articles, and from these, 27 were review and meta-analysis articles, hence, excluded. From the broader search, about 266 articles were excluded while screening for abstracts, and only eight articles were eligible. From database and register searches, 221 articles were retrieved after removing duplicates and abstract screening. From these, 53 articles were screened for full text, and 37 articles were removed further due to irrelevant outcomes, being out of scope, and being conducted on different target populations. Finally, a total of 17 articles were included in the narrative review, and 15 articles were used for the pooled meta-analysis (Fig 1).

### Characteristics of included studies

For this review, a variety of observational studies assessed the association between different levels of khat consumption and nutritional status. Hence, a total of seventeen studies involving 45,679 subjects were included in the current review paper. With regard to the study design, the majority (n = 11; 71% of studies) were analytical cross-sectional studies, except for three case control studies (17.4%), and three of them were comparative cross-sectional study types (17.4%). Related to this, a large number of studies were conducted among the general adult population, prisoners, HIV patients, tuberculosis patients, and one study among cobblestone workers. As the intention of the study is to assess the effects of Khat on nutritional status and the included studies make efforts to control potential confounders, studies on this target population were considered eligible. About sixteen studies were published, except one, which was unpublished comparative work yet relevant to the current review (Table 1).

**Table 1:** Characteristics of included studies investigating the relationship between khat chewing and undernutrition among adults in Ethiopia.

S. No	Author, Year	Method Summary	Main Findings	Reference
1	Gelan Z et al., 2023 [19]	*A mixed method study on randomly selected 297 CSWs in Hawassa city *Used BMI to assess Nutritional status *Multinomial model was used	*14.1% and 16.8% of them were underweight and over nourished, respectively *Chewed Khat regularly (AOR = 0.23) drugs regularly (AOR = 10.57) increase the odds of underweight *16.8% of them were HIV positives	[19]
2	Weldehaweria N.B. et al., 2017 [36]	*A matched case-control study design was conducted * study targets PLHIV on ART (n = 342). *Cases were selected by simple random sampling and controls *Conditional logistic regression was used to compute relevant associations by STATA version 12.	*Ever khat chewing is associated with reduced risk of undernutrition *Alcohol consumption and other dietary factors were associated with malnutrition	[36]
3	Tesfaw et al. 2021 [37]	* A national survey analysis (DHS 2016 survey data) *Separate analysis for men and women was done *10,245 individuals *Logistic regression was done	*23.8% of men and 25.3% of women are undernourished *Poor men had higher odds of undernutrition *khat is associated with undernutrition (AOR = 1.162 (0.974, 1.382); p = 0.0625) among men and women (COR = 0.63; p = 0.020)	[37]
4	Adem et al. 2023 [38]	*A facility-based case control study * 113 cases and 113 controls *nutritional status was assessed using MUAC below 23 cm (cases) and control otherwise	*Upon adjustment for confounder variables, at least one substance use increases the odds of undernutrition among lactating women by 3.65 times *The study did not report Khat chewing as separate exposure	[38]
5	Damie et al. 2015 [39]	* A community-based cryosection study on 319 adolescents *Underweight was measured using weight and height (BMI for age) *Logistic regression was employed	*Khat chewing could increase risk of underweight (AOR = 2.45; 1.07, 5.64)	[39]
6	Abera et al. 2017 [20]	•Underweight (Defined as BMI below than 18.5 Kg m-^2^) *A survey of 809 prisoners were done *	*Prevalence of underweight was 25.2% (95% CI; 22.3%- 28.3%). *Khat Chewing (OR = 2.08; 95% CI = 1.17, 3.70) increase the risk of underweight	[20]
7	Assefa et al. 2020 [18]	* Facility-based cross-sectional study *530 psychiatric patients were included *Anthropometric measurements were done *BMI was calculated and defined *Multinomial logistic regression was employed	*20% of adults were undernourished *chewing khat (OR = 0.97) for undernutrition *Not Chewing Khat (AOR: 3.92, 95% CI: 1.63–9.42) were associated with higher risk of overnutrition *Not involved in physical activity (AOR: 2.98, 95% CI: 1.37–6.49) *Not consuming alcohol (AOR: 0.20, 95% CI: 0.56–0.74) for overnutrition	[18]
8	Hussien B. et al. 2019 [16]	* Facility-based survey of new tuberculosis patients was conducted *BMI was calculated *Logistic regression was done	*Prevalence of nutritional deficiency was 63.2%. * Khat chewing (AOR = 2.32(1.18–4.35) is associated with undernutrition	[16]
9	Belew M. et al. 2000 [15]	*A survey of 1200 adults were conducted in 1997 * setting was rural community in Ethiopia	* Khat chewing was more common among Muslim, males, and youths as compared to others * Khat use (AOR = 1.76, 1.24–2.48 was associated with undernutrition among adults	[15]
10	Oumer et al. 2019 [26]	* A survey of 333 adult HIV patients were survey *Nutritional status was the outcome *History of substance uses including Khat was assessed.	*History of substance use was associated with undernutrition (AOR = 1.84 (1.09, 3.08) *23.7% of them were undernourished based on BMI cutoff point	[26]
11	Dedha et al. 2017 [40]	* A facility-based survey of HIV positive individuals *study setting: Eastern Ethiopia (Oromia region) * BMI less than 18.5 was used to identify undernutrition *Logistic regression was employed	*Khat chewing (AOR = 0.589, 95% CI: 0.377, 0.92) * 30% of the clients were diagnosed with undernutrition	[40]
12	Assefa et al. 2023 [25]	* a case control study of 93 and 186 cases and controls was conducted * Underweight was defined as having BMI below 18.5 kg m^-2^ *Data were collected from April to May 2022	*khat chewing (AOR = 3.36; 1.20,11.3) were determinants of underweight*	[25]
13	Kabtu et al. 2022 [17]	*Institution-based cross-sectional study was conducted in 2022 *Among 414 adults on antiretroviral therapy. *The data were collected by interview, record review and physical measurements. *Multinomial logistic regression was used	*Khat (AOR = 2.78(1.09–7.114)) and drinking alcohol (AOR = 1.61(1.09–3.61) were associated with undernutrition	[17]
14	Minas et al. 2022 [22]	* A comparative cross-sectional study was conducted * 446 (223 in khat chewer and non-chewer group) were involved *Nutritional status based on body mass index (kg/m2).	*Khat chewers (39.0%; 32.8–45.6) than non-chewers (22.4%; 17.4, 28.4; p < 0.0001). * Chewing daily (AOR = 3.14, = 1.08, 9.15) were associated with undernutrition compared to less frequent chewing among chewers	[22]
15	Kassie et al. 2020 [41]	* Analysis of subsample of DHS data for 2016 was done * A sub sample of 2848 women in reproductive age was conducted	* 17.6% of them were undernourished where 78.3% of them were from rural areas. *non-khat chewers women had a higher risk of undernutrition (AOR = 1.51 (1.02, 2.23)	[41]
16	Moges et al. 2016 [42]	* a survey of cobble stone workers was conducted (n = 422) in Addis Ababa A systematic random sampling was done across three project sites *Anthropometrics were done to calculate BMI * Logistic regression was done	* Khat chewing could increase the risk of undernutrition by two-folds (AOR = 1.69 (0.90–3.17) *Similarly, alcohol drinking (AOR = 3.32 (0.80–6.11) and cigarette smoking (AOR = 2.02 (1.06–3.87) increase risk of undernutrition	[42]
17	Hailesellasie G. et al. 2023 [43]	* A comparative survey among khat chewer and non-chewer adults were conducted *Targets were randomly selected adult males *Logistic regression analysis *The outcome was nutritional status diagnosed by BMI	*A total of 78 (32.0%) khat chewers and 60 (23.9%) of non-khat chewers were undernourished. *Khat chewing (AOR = 1.60; 1.04–2.45) increase risk of undernutrition	[43]

Concerning the outcome definition, almost all of the included studies assessed nutritional status using the WHO BMI classification, where a value below 18.5 kg/m^2^ was used to ascertain undernutrition or underweight. However, one included study used a MUAC cutoff point below 23 cm to define malnutrition. These might not make a huge difference, as MUAC could predict BMI when BMI measurement is not appropriate. In addition, we conducted a sensitivity analysis by adding and removing this study if it had a significant impact on the pooled effect sizes (Table 1).

However, there are some variations in defining exposure (khat chewing). As khat chewing can be characterized in terms of duration, intensity, and frequency, some limited studies tried to report disaggregated exposure levels by intensity, duration, and frequency [15]. Since the majority of the studies assessed and reported khat chewing status as a current chewer and history of khat chewing, we decided to make the definition of khat chewing more comprehensive by including current chewing status and past history (Table 1).

The included studies employed a range of samples, with a minimum of 226 in the survey [38] to a maximum of 10,245, based on the reanalysis of the DHS 2016 data for women in the reproductive age group [37]. When we disaggregate studies by region, the majority of studies were from Oromia (n = 5), SNNPR (n = 3), Amhara (n = 3), Addis Ababa (n = 2) and Tigray (n = 1). Three studies (n = 14,293 participants) [15,37,38] were conducted at a national level in multicenter mode (Table 1).

### Risk of bias assessments

The risks of bias for each study were evaluated for a cross-sectional study and a case-control study separately. We employed the eight items for survey designs and the 10-item checklist for case control studies as well. The details of risk bias for individual studies are included in S1 and S2 Tables. The majority of the studies were considered to have a low to moderate risk of bias. This was mainly due to an unclear definition of exposure (khat consumption), where some studies assessed khat chewing level from multiple perspectives [15,37]. One study reported khat chewing in the context of current substance use, where the variable measurement could be biased [26]. This was also the same problem in defining khat consumption with the case control studies as well [25,36,39]. The certainty of the evidence was evaluated with a moderate level of recommendation for khat consumption and undernutrition risk in Ethiopia.

### Association between khat consumption and undernutrition

**Fig 2:**
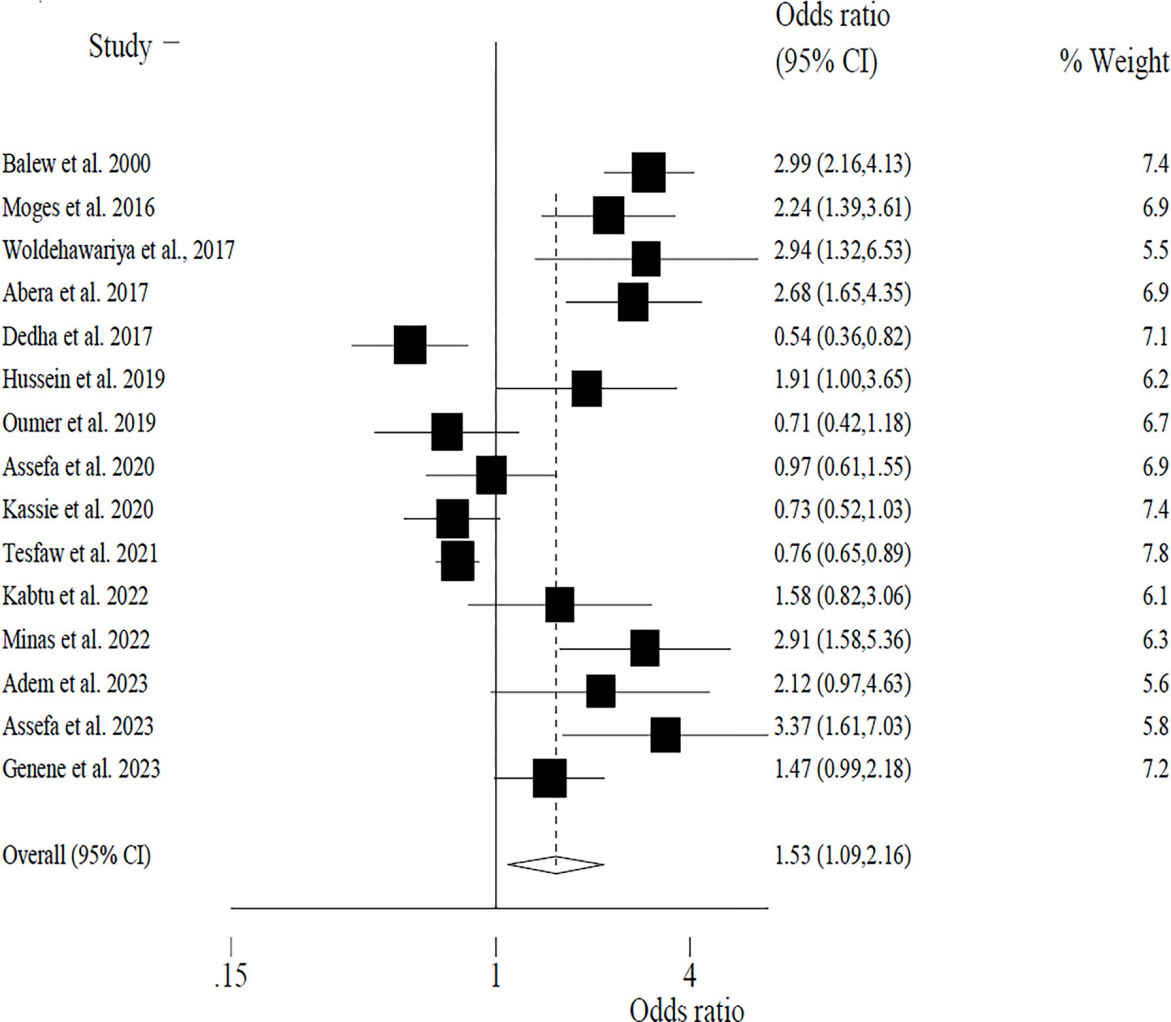
Forest plot showing the pooled effects of khat consumption on the risk of undernutrition in Ethiopia.

A total of fifteen articles were included in the pooled effect size estimates, and two articles were not included due to a lack of evidence on each cell value. From the individual study results, four studies showed that khat chewers had lower odds of being underweight as compared to non-chewers [18,26,37,40]. We found significant heterogeneity (X^2^ = 132.8; d.f. = 14; p = 0.0001). In addition, the estimate of between-study variance (Tau-squared) was not statistically significant (p = 0.381), indicating less heterogeneity between included studies. Overall, khat chewing had been shown to significantly increase the risk of undernutrition by 53% (pooled OR = 1.53; 95% CI: 1.09–2.16; p = 0.014). The weight given to each study is presented in Fig 2. Under the fixed effect model with due weight to studies with a larger sample size, khat chewing could increase the odds of undernutrition by 12% (1.12; 95% CI: 1.02–1.23). In this model, the major study weight was 49% [37] and 10% [41], which indicated that the random effect model was a more reasonable estimate than the fixed effect model (Fig 2).

### Publication bias

**Fig 3:**
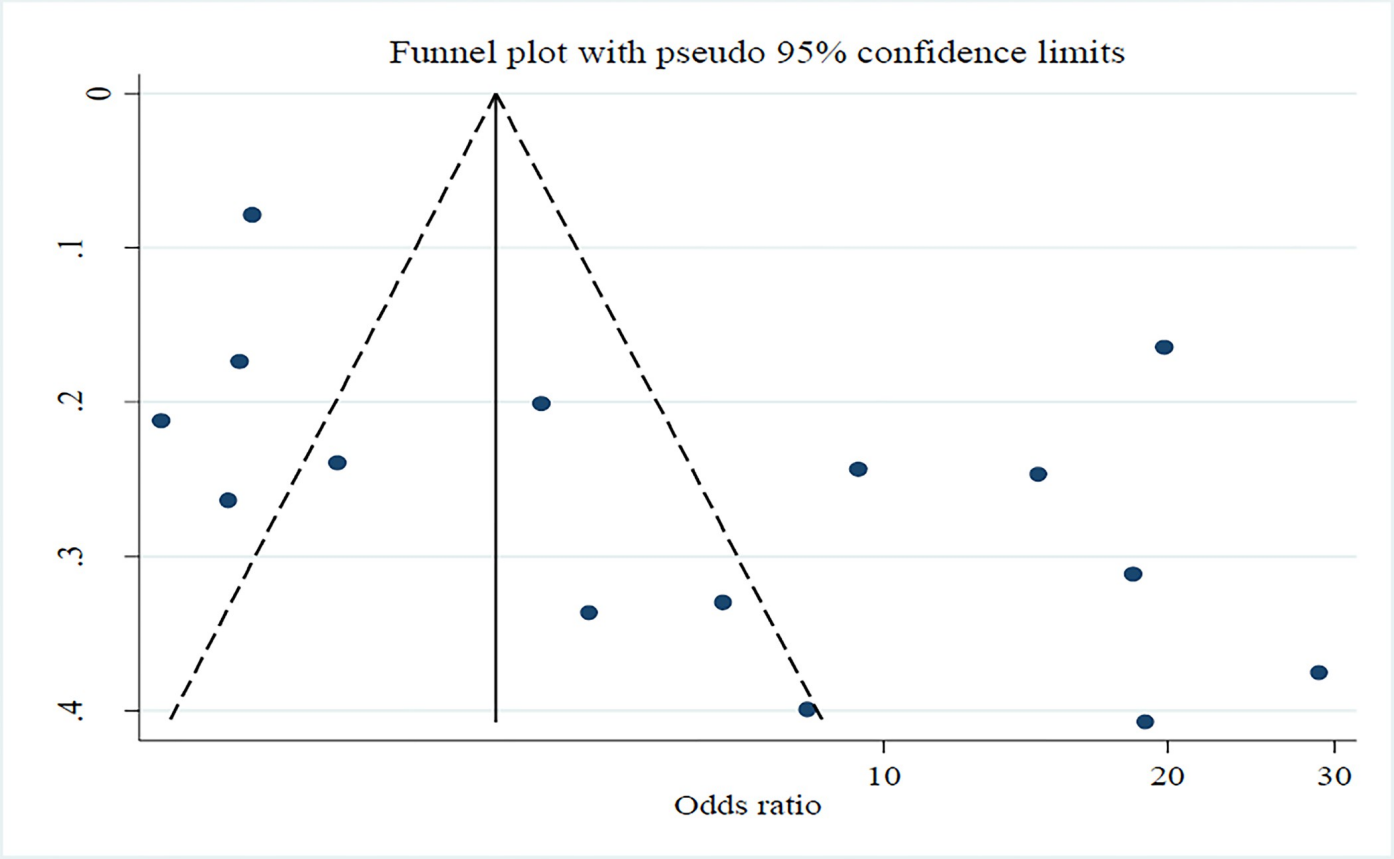
Funnel plot showing the risk of publication bias by plotting odds ratio against the standard error for the odds ratio in Ethiopia.

The risk of publication bias was evaluated using a funnel plot and the odds ratio as a function of the standard error of the effect size. This is clearly indicated in Fig 3, where the risk of publication bias was found to be high. However, this might be due to the fact that the three national-level studies had a relatively high number of samples (n = 14, 293), where 10,245, 2848, and 1200 samples were included [15,37,41], and these studies might have a higher effect with a lower standard error of the estimate as compared to studies with a lower sample size. The results of five studies were within the triangle, and the estimates of six studies were pretty close to the triangle. Hence, the inherent study heterogeneity including the national level studies and local studies could have created increased risk of publication bias.

### Sensitivity analysis

Sensitivity analysis helps to evaluate the robustness of the findings and provides insights into the potential influence of individual studies on the overall results. Hence, the impacts of removing each study from the meta-analysis were assessed. Table 2 provides information on the pooled odds ratios, measures of study heterogeneity, and p-values for each study when it was excluded. Notably, the results indicate significant heterogeneity among the studies, as evidenced by the calculated chi-squared and tau-squared values. Overall, there is a significant association between khat consumption and the risk of undernutrition among adults in Ethiopia (Table 2).

**Table 2:** Sensitivity analysis (n = 14 studies) after removing each study from the meta-analysis for association between khat consumption and risk of undernutrition in Ethiopia.

S.No	When each studies removed	No of subjects	Pooled odds ratio	Measures of study heterogeneity	p-value
**1**	Weldehaweria et al., 2017 [36]	19081	1.48(1.04–2.09)	X^2^ = 127.1: p = 0.0001 Ʈ^2^ = 0.375	0.029
**2**	Tesfaw et al. 2021 [37]	9178	1.63(1.14–2.31)	X^2^ = 90.5: p = 0.0001 Ʈ^2^ = 0.373	0.007
**3**	Adem et al. 2023 [38]	19197	1.51(1.06–2.14)	X^2^ = 130.2: p = 0.0001 Ʈ^2^ = 0.385	0.023
**4**	Abera et al. 2017 [20]	18614	1.47(1.04–2.08)	X^2^ = 119.9: p = 0.0001 Ʈ^2^ = 0.366	0.030
**5**	Assefa et al. 2020 [18]	18893	1.59*1.10–2.29)	X^2^ = 132.4: p = 0.0001 Ʈ^2^ = 0.410	0.013
**6**	Hussien et al. 2019 [16]	19051	1.51(1.06–2.16)	X^2^ = 130.1: p = 0.0001 Ʈ^2^ = 0.390	0.023
**7**	Belew et al. 2000 [15]	18223	1.44(1.04–1.99)	X^2^ = 94.2: p = 0.0001 Ʈ^2^ = 0.301	0.026
**8**	Oumer et al. 2019 [26]	19090	1.62(1.13–2.32)	X^2^ = 129.5: p = 0.0001 Ʈ^2^ = 0.395	0.008
**9**	Dedha et al. 2017 [40]	18986	1.66(1.17–2.36)	X^2^ = 120.1: p = 0.0001 Ʈ2 = 0.375	0.005
**10**	Assefa et al. 2023 [25]	19126	1.46(1.03–2.06)	X^2^ = 124.1: p = 0.0001 Ʈ^2^ = 0.367	0.032
**11**	Kabtu et al. 2022 [17]	19009	1.53(1.07–2.19)	X^2^ = 131.7: p = 0.0001 Ʈ^2^ = 0.394	0.019
**12**	Minas et al. 2022 [22]	18997	1.47(1.04–2.08)	X^2^ = 123.25: p = 0.0001 Ʈ^2^ = 0.368	0.031
**13**	Kassie et al. 2020 [41]	16575	1.63(1.13–2.18)	X^2^ = 126.0: p = 0.0001 Ʈ^2^ = 0.412	0.009
**14**	Moges et al. 2016 [42]	19001	1.49(1.05–2.12)	X^2^ = 124.5: p = 0.0001 Ʈ^2^ = 0.382	0.027
**15**	Hailesellasie et al. 2023 [43]	19921	1.54(1.07–2.23)	X^2^ = 130.9; p = 0.0001 Ʈ^2^ = 0.417	0.021

### Subgroup analysis

The disaggregated estimates are done by region, population type, and study design and are presented in Table 3, which shows a positive association with the risks of undernutrition but is not statistically significant. When disaggregated by region, the heterogeneity between studies was insignificant except for two studies in Addis Ababa (urban setting), where khat chewing is significantly associated with undernutrition (pooled OR = 1.77; 95% CI: 1.17–2.68). Among high-risk population segments, khat is associated with a 47% increased risk of undernutrition with small between-subject variations (p = 0.43). However, based on the three case control studies with a better quality of evidence, it was found that khat chewing is significantly associated with undernutrition (pooled OR = 2.78; 95% CI: 1.78–4.33). Overall, studies using large samples from national level analysis showed an averaged effect (lower odds ratio) compared to studies reported from Oromia(OR = 1.80), Addis Ababa (OR = 1.77), and Amhara (OR = 151). These could imply that the risk of undernutrition could be higher in a certain setting owing to many factors (Table 3).

**Table 3:** Subgroup analysis for the effects of khat chewing on the risk of undernutrition by region, population, and study design of included studies in Ethiopia.

Factors	Options	Pooled odds ratio	Heterogeneity	P-value
**By region**	Amhara (n = 2)	1.51 (0.60–4.37)	X^2^ = 8.76 (d.f. = 1) p = 0.003 Tau-squared = 0.46	0.349
	Oromia (n = 5)	1.80 (0.82–3.97)	X^2^ = 32.96 (d.f. = 4) p = 0.0001 Tau-squared = 0.71	0.145
	SNNPR (2)	1.03(0.47–2.27)	X^2^ = 3.58 (d.f. = 1) p = 0.058 Tau-squared = 0.24	0.943
	Addis Ababa (n = 2)	1.77(1.17–2.68)	X^2^ = 1.80 (d.f. = 1) p = 0.180 Tau-squared = 0.04	0.007
	National (n = 3)	1.18 (0.51–2.72)	X^2^ = 58.4 (d.f. = 2) p = 0.0001 Tau-squared = 0.52	0.679
**By population**	General population (n = 7)	1.61 (0.96–2.70)	X^2^ = 86.94 (d.f. = 6) p = 0.0001 Tau-squared = 0.43	0.071
	High-risk population (HIV, commercial sex workers, psychiatry and TB patients) (n = 8)	1.47 (0.89–2.44)	X^2^ = 44.5 (d.f. = 7) p = 0.000 Tau-squared = 0.43	0.130
**By study design**	Cross sectional study (n = 12)	1.35 (0.94–1.95)	X^2^ = 115.42 (d.f. = 11) p = 0.000 Tau-squared = 0.36	0.101
	Case control study (n = 3)	2.78 (1.78–4.33)	X^2^ = 0.75 (d.f. = 2) p = 0.689 Tau-squared = 0.00001	0.0001

Finally, a separate meta-regression was conducted to evaluate the effects of the potential factors on the heterogeneity of the effect size. Hence, region, study design, sample size, and population type were conducted, and these variables did not show significant effects on the overall study heterogeneity (p-value > 0.05).

## Discussion

Due to the widespread consumption of the substance khat [8,9] and prevailing malnutrition in the country [44,45], there was a need for clear evidence on the association between these two to support existing nutrition interventions. Moreover, this piece of evidence could help the country move towards achieving the sustainable development goals and bring about the intended economic growth through various approaches. The association between khat chewing and undernutrition has been a subject of interest in Ethiopia, where khat consumption is prevalent. Khat, a psychoactive plant native to the Horn of Africa, contains several alkaloids, including cathinone and cathine, which possess amphetamine-like properties [12,13,46]. The stimulating effects of khat, including increased alertness and reduced fatigue, have made it popular among adults in Ethiopia. However, the long-term consequences of khat chewing on health outcomes, including undernutrition, have raised concerns.

Hence, the present meta-analysis aimed to explore the association between khat chewing and the risk of undernutrition among adults in Ethiopia. Our findings, based on a pooled analysis of 17 articles, revealed a significant association between khat chewing and an increased risk of undernutrition, with a pooled odds ratio of 1.53 (95% CI: 1.09–2.16). Thus, our findings suggest that khat chewing is associated with an increased risk of undernutrition. This observation is consistent with the existing literature, which has reported similar associations. For instance, a study by Tesfaye et al. (2018) found that khat chewing was significantly associated with a higher prevalence of underweight among adults in Ethiopia [47]. Another study by Ahmed et al. (2019) reported a positive association between khat chewing and a higher risk of malnutrition in a rural community in Ethiopia [23]. Our meta-analysis further strengthens these findings by providing a pooled estimate of the association across multiple studies. This burden is still huge among late adolescents and youths [48].

The underlying mechanisms linking khat chewing and undernutrition are likely multifactorial [15,49]. One possible explanation is the appetite-suppressing effects of khat. Cathinone, one of the psychoactive components of khat, acts as a central nervous system stimulant and may reduce appetite and food intake [12,13,15,46]. Prolonged khat chewing may lead to decreased caloric intake and inadequate nutrient intake, thereby increasing the risk of undernutrition [18]. Moreover, tannins and dietary fibers, the anti-nutritional factors found in many vegetables and khat are known to reduce mineral and other nutrient absorption, especially for iron, zinc and calcium bringing additional risks of micronutrient deficiency [44,50,51]. These micronutrient deficiencies could further increase the risk of macronutrient undernutrition in various ways [44]. A strong association between maternal khat use [52] and the neonatal outcomes and body composition [53] of adults were also reported so far.

The long-term habit of khat chewing may predispose individuals to oro-dental problems, limiting diversified dietary consumption [54]. More importantly, these could be associated with long-term exposure to pesticide and insecticide residues accumulating over time. These could further limit nutrient absorption and metabolism [55] and susceptibility to chronic illness, aggravating the risk of undernutrition. More specifically, exposure to chemicals like DDT, malathion, and other potentially hazardous chemicals could be worse [56,57]. Additionally, those who chew khat usually have a habit of using other substances like alcohol, *shisha*, cigarette, and others, further limiting food intake and predisposing to malnutrition [15,58,59].

Studies have indicated that habitual khat chewing is associated with a lower diversified diet, reduced appetite, and decreased meal frequency and amount, which may lead to negative nutritional outcomes, particularly among individuals engaged in physically demanding activities [60,61]. However, the impact of khat on nutrition and health is complex, with potential positive and negative consequences depending on individual factors and contextual circumstances. Prolonged khat chewing may also be linked to increased consumption of high-calorie soft drinks and sedentary behavior, potentially contributing to the growing issues of obesity and non-communicable diseases in urban areas [62].

On the contrary, some of the previous studies showed an inverse association between khat consumption and the risk of undernutrition [25,26]. These studies were mainly conducted among diseased individuals (tuberculosis, HIV, and psychiatric disorders), where khat consumption is restricted due to various medical indications. Hence, those with advanced illnesses tend to reduce or avoid khat consumption frequency, intensity, and duration compared to those with stable clinical conditions. Due to this and other potential situations, the risk could be higher among non-khat-chewers. Furthermore, as indicated before, khat chewing with longer hours of stay in a sedentary lifestyle could limit physical activity and may increase the risk of obesity on the reverse. However, khat chewing on any of the above premises, is not beneficial in promoting optimal nutrition and economic development.

More importantly, khat chewing may also contribute to undernutrition indirectly through its social and economic impact. Khat chewing is often associated with social gatherings and has been reported to divert financial resources away from basic necessities, including food [63]. This diversion of resources may result in reduced food security and inadequate access to a balanced diet, ultimately leading to undernutrition. This could further limit economic productivity and working hours, limiting economic growth, food security, and self-sufficiency for better nutrition and health [6,64].

The disaggregated estimates showed heterogeneity among studies by setting, population and study design. Among high-risk population segments, khat is associated with a 47% increased risk of undernutrition with small between-subject variations (p = 0.43). However, based on the three case control studies with a better quality of evidence, it was found that khat chewing is significantly associated with undernutrition. Overall, studies using large samples from national level analysis showed an averaged effect (lower odds ratio) compared to studies reported from Oromia, Addis Ababa, and Amhara regions. These could imply that the risk of undernutrition could be higher in a certain setting oning to many factors. Furthermore, the negative impacts of khat chewing might concentrate in certain regions and populations that could be associated with the food security, feeding practice and other concomitant substance uses.

It is important to acknowledge some limitations of the current study. Firstly, the included studies were observational in nature, which limits our ability to establish causality and the association could be further confounded by many unmeasured factors. Secondly, there was heterogeneity among the studies in terms of study design, sample size, population characteristics, and the risk of bias among the included studies. However, we performed a random-effects model to account for this heterogeneity. Finally, the studies included in this meta-analysis were conducted in Ethiopia, which may limit the generalizability of our findings to other populations yet very informative for the study setting.

In conclusion, the current meta-analysis provides evidence of an association between khat chewing and an increased risk of undernutrition among adults in Ethiopia. These findings highlight the need for public health interventions to address the potential adverse effects of khat chewing on nutritional status. Efforts should focus on raising awareness about the potential health consequences of khat chewing, improving access to nutritious food, and promoting healthier alternatives to cope with fatigue and stress. Thus, it is imperative to consider khat chewing as relevant risk factors in addressing undernutrition and its consequences in Ethiopia. Further rigorous research shall be conducted in a more controlled and well-designed manner to empirically illustrate the role of khat chewing for undernutrition and overnutrition.

## Conclusion

Khat chewing poses a significant risk for undernutrition, especially among women and vulnerable groups like HIV, tuberculosis, and psychiatric patients. This risk likely increases with higher intensity and prolonged duration of chewing, hence, high-intensity and prolonged khat chewing may significantly exacerbate the risk of undernutrition. Multifaceted agricultural, social, and economic interventions are required to halt the existing khat consumption, especially among the vulnerable segments of the population like HIV, tuberculosis, and psychiatric patients. Additionally, khat chewing’s link to overnutrition and non-communicable diseases might be associated with increased physical inactivity, high-calorie, and processed consumption. Further research on the association between khat chewing and adult undernutrition in Ethiopia should prioritize longitudinal studies to establish causality and investigate specific mechanisms. Additionally, exploring the potential long-term effects of pesticide residues and examining the link between khat chewing and overnutrition/non-communicable diseases will provide valuable insights for targeted interventions and policies to improve nutritional health outcomes. Hence, public health interventions are needed to address the negative effects of khat chewing on nutrition, including raising awareness, promoting healthier alternatives, and implementing multifaceted agricultural, social, and economic interventions at a scale.

## Supporting information

S1 Checklist PRISMA 2020 checklist. (DOCX)

S1 Table The JBI parameters for inclusion of cross-sectional study articles on the association between khat chewing and undernutrition among adults in Ethiopia. (XLSX)

S2 Table The JBI parameters for inclusion of case control study articles on the association between khat chewing and undernutrition among adults in Ethiopia. (XLSX)

## Abbreviations

AOR: Adjusted Odds Ratio
BMI: Body Mass Index
GRADE: Grading of Recommendation Assessment Development and Evaluation
MeSH: Medical Subject Heading
PRISMA: Preferred Reporting Items for Systematic Review and Meta-analysis
WHO: World Health Organization
